# The Unmet Needs of Parents of Highly Dependent Children with Cerebral Palsy

**DOI:** 10.3390/ijerph16245145

**Published:** 2019-12-16

**Authors:** Nor Azlin Mohd Nordin, Eyu Hui Shan, Asfarina Zanudin

**Affiliations:** Physiotherapy Programme, Center for Rehabilitation and Special Needs, Faculty of Health Sciences, Universiti Kebangsaan, Jalan Raja Muda Abdul Aziz, Kuala Lumpur 50300, Malaysia; norazlin8@ukm.edu.my (N.A.M.N.); shan0206@hotmail.com (E.H.S.)

**Keywords:** cerebral palsy, children, parents, family needs, unmet needs

## Abstract

The overall care for children with cerebral palsy (CP) is challenging to the family which causes significant impacts to their livelihood. There is limited qualitative research that reports the unmet needs of parents with physically disabled children, especially highly dependent CP. The aim of this study was to explore the unmet needs of parents of highly dependent children with CP. A qualitative study using semi-structured face to face interviews was carried out among nine parents of children with CP with gross motor classification function score (GMFCS) levels III, IV, and V. The interviews were tape-recorded and transcribed verbatim. Transcribed data was analysed using thematic analysis method. Several unmet needs were highlighted by the parents; namely the needs in receiving information regarding CP conditions, getting psychological and financial support and explaining the child’s condition to strangers. In addition, parents expressed the need for better support from the social welfare department, as well as in effectively organising family functioning. The findings of this study indicate that there is a need for the healthcare professionals to develop suitable strategies to assist the parents of highly dependent children with CP in fulfilling their specific needs. The role of relevant agencies should be optimised in addressing this area of concern.

## 1. Introduction

Taking care of children with cerebral palsy (CP) is challenging to the family. It commonly causes significant psychosocial distress and adverse effects to their quality of life [1]. Knowing the family’s needs will ease their burden and assist in overcoming their ambivalence. This is one of the important features of family-centred care, which to date is known to be the best practice in pediatrics rehabilitation, resulting in many positive outcomes [2]. Family-centred care is an approach that actively acknowledges and addresses family members’ concerns related to their children’s or sibling’s condition. Healthcare professionals play roles in helping these children and their families beyond their clinical expertise by assessing the unique strength and needs of the families [2].

The needs of a family of children with CP are different depending on the level of the child’s functional ability, which is usually classified using Gross Motor Functional Classification System (GMFCS) [3]. Children with GMFCS levels IV and V, i.e., severely disabled or highly dependent group, require additional health care resources, higher cost of medications, and are presented with more frequent medical problems than those with GMFCS levels I, II and III [4]. Previous studies showed that the family of children with CP who are severely disabled are more likely to have greater needs [5,6,7,8,9] than family with less disabled children.

Previous studies which assessed the needs of family of children with CP or disability have reported several main needs which include the need for information about the condition of their children, treatment options, presently available services and the services they may receive in the future [6,7,8,9]. Additionally, these families require additional support, community services and financial aid for therapy and special equipment [6,7,8]. Two Malaysia-based studies investigating the needs of caregivers of children with disabilities using questionnaires (i.e., 20-item Caregiver Needs Scale and Family Needs Survey) reported that caregivers, particularly those who have children with multiple disabilities, needed more assistance in getting information and require support in coping with the burden of care [10,11]. However, these previous studies involved caregivers with children with not just physical disabilities such as CP but other conditions such as learning, vision and hearing problems, hence limiting the interpretation of the findings.

To date, previous studies on the topic of family needs of children with CP or disability have been conducted using a quantitative approach with the use of questionnaires [6,7,8,9,10,11,12,13]. This approach limits the retrieval of information from the parents as they are restricted in expressing their thoughts and opinion in a more detailed way. Due to the complexity of needs among the family of highly dependent CP, qualitative research is required to explore this area of concern and to enable effective planning of family-centred care for this population. This is based on the Behavioral Model of Health Services, a multilevel model that incorporates both individuals and contextual determinants with three major components which are predisposing factors, enabling factors and needs factors [5] that leads to utilization of health services.

To the best of our knowledge, only one qualitative study has been conducted which focused on the experience of family to young children with CP. However, the needs of the families explored in the study were mainly needs related to physical and occupational therapy for their children [14]. Hence, the aim of this study was to further explore the unmet needs of parents of highly dependent children with CP in daily living which reported in previous quantitative studies by using the qualitative study approach.

## 2. Materials and Methods

### 2.1. Study Design

The study was based on a semi-structured face to face interview to explore the unmet needs of parents of highly dependent children with CP. This was carried out in the physiotherapy clinic, Faculty of Health Sciences, Universiti Kebangsaan Malaysia. This study obtained ethical approval from the Universiti Kebangsaan Malaysia Research Ethical Committee (study code NN-2017-049).

### 2.2. Participants

The participants of this study were selected from among parents of children with highly dependent CP receiving physiotherapy at the clinic. Highly dependent is defined as significantly needing somebody or something in order to survive or be successful [15] and identified using GMFCS. The participants who agreed to participate were given a date for the interview session which occurred in a comfortable, quiet room of the clinic.

The recommended sample size to achieve data saturation in an interview is 12 [16]. In this study, a total of 15 parents who met the main inclusion criteria, i.e., having children with highly dependent CP, were approached following screening of the patients’ database of the clinic. However, of these, only nine parents agreed to voluntarily participate in this study and were enrolled. The remaining six parents could not participate due to time constraints. The nine parents then were asked to decide who the main caregiver is, since only the main caregiver would be recruited. All recruited participants provided informed consent prior to participating in the study.

### 2.3. Interview Process

Conducting in-depth interview involves six stages which are thematising, designing, interviewing, transcribing, analysing and verifying [17]. Because this study was intended to further explore the needs of family of children with CP, an interview protocol was decided based on the Family Needs Survey (FNS) which contains 35 items covering six types of needs, namely information, support, explaining to others, community, financial and family functioning [18]. The interview protocol was pilot-tested before the actual interviews to ensure clarity of the semi-structured questions and to estimate the duration needed for the actual interview.

Each interview was carried out using a discovery-oriented method which allowed deep exploration on the respondent’s points of view and took 45 to 60 min. The interviews were conducted in Malay language as preferred by all the parents. The researcher (E.H.S) facilitated the participants to express their thoughts and opinions by using prompts. All interviews were visually and audio-recorded upon the agreement of the participants. Concurrently, a research assistant took field notes during each interview, further ensuring triangulation during data collection. Data saturation was considered reached when no new ideas were expressed and there were repetitions of ideas among the participants [19].

### 2.4. Data Analysis

All interview data was transcribed verbatim. The transcripts were then translated into English language using back-to-back translation approach, following which the transcripts were analysed using the thematic analysis method. Triangulation in data analysis was ensured by getting two researchers to independently read and interpret the transcripts [20,21]. The two researchers then identified relevant quotes which fulfill the intended themes or categories by constantly reading and re-reading the transcripts. Interpretations made by the two researchers were then compared and discussed until reaching an agreement. The two researchers also agreed upon quotations from participants that best represent each of the agreed themes.

## 3. Results

### 3.1. Participants’ Profile

The nine enrolled participants ranged from 32 to 51 years of age (mean = 37.89; SD = 5.88) and eight of them are female (88.89%). Table 1 shows the profile of parents and their children with CP who participated in this study. All participants live together with their spouse and other children who are the CP child’s siblings. All children with CP whose parents were involved in this study are able to follow simple command with the exception of one child (P2).

### 3.2. Interview Findings

Several unmet needs related to knowledge and information, social, welfare and financial support, ability to explain child’s condition to others and family functioning were highlighted by the parents.

#### 3.2.1. Receiving Information Related to the Child’s Condition

The majority of the parents stated that they were deprived of information related to their child’s condition particularly in the early phase post-diagnosis. Their main source of information was from the internet, although they wished the healthcare providers had provided comprehensive information during their visit to the hospital or healthcare centers. Three parents (P1, P4, P7) took the initiative to validate the information obtained from internet with their healthcare providers. Other than the internet, parents of other children with CP were also one of their main sources of knowledge, especially information on ways to handle their child. A few parents (P2, P3, P9) perceived the experience-based knowledge as very helpful.

*“Ok, for now I am very disappointed because our primary healthcare centre does not provide much information and I’m forced to read. I had to search for those information [myself].” (P1)*

*“For information … we have to search ourselves. I always study on the internet myself then I will ask the doctor during our appointment. The doctor gave a small book regarding CP … like a leaflet … Then from there we searched on the Internet. So, most of our information is from the Internet. Some of the information we received from … other parents’ experience … shared among parents.” (P7)*

*“Everything I know is from the Internet. I type “CP” then all information comes out … There is more information from the Internet than from the hospital staffs.”* (P4)

*“The doctor speaks in English and I don’t understand. I see other families of children with CP … some of them cannot understand … like English … we have language problem because most information is in English right? That’s why I learn more from other parents” (P8)*

*“I received most information from other parents’ experience…” (P2)*

*“The session … we call as [a] group, … and we discuss with other parents. We … ask for their opinion and … ask how they handle [their child].” (P3)*

*“For now … I ask friends [other parents] whose child is like mine…” (P9)*

#### 3.2.2. Social Support

The parents expressed that they received adequate social support from people surrounding their lives such as parents, siblings, friends and employers who have been understanding and supportive.

*“Family … my siblings are very … understanding and they help … sometimes I am forced to work away … need to be outstationed … so they help me in [sending my child to] appointment. My employer too … he (supports) … it is very important for me to talk to the head of department and then to my colleagues. So…when…in terms of management or other things … working schedule … I am able to negotiate … with friends.” (P3)*

*“No problem. My parents support a lot. All are caring … Alhamdulillah most of my friends are too … ah … what … they have exposure to this kind of knowledge. So they understand.” (P1)*

*“Sometimes … I have a conflict with my parents. They won’t agree if I wish to bring him to other places for treatment. So … will need to explain to them. They will support eventually although they don’t really agree.” (P6)*

When asked to describe the support from the Government office, some parents claimed that the support from the social welfare department could be improved as currently it is limited to just daily consumables and disposables for the child. Parents have difficulty in receiving funds to buy special equipment for their child. The support was also very slow and not readily available. Most of the parents took their own initiative to approach the social welfare department for assistance such as to locate day care centre for their child. Access to special schools for the children is also difficult and some parents had to send their child to private school for education.

*“Not given (any help) at all. Any help … or anything … nothing. But they gave pampers … er … gave milk. Exactly … where do we get (sponsorship for) special equipment? Nothing, couldn’t and didn’t buy anything.” (P8)*

*“Never received. Never received if not requested. We need to request ourselves, we need to apply and prepare a letter ourselves, then only we are able to receive. It has been 2 years and we haven’t received (reply). Now, I am hoping for a school which will be able to help them. Meaning to say … the school fees aren’t expensive so we could afford it.” (P6)*

*“Received welfare like milk … pampers … because his milk is special milk, I can’t afford to buy. But other support … That I think should be available … supposedly special school for CP. Actually I got here by my own research … I tried asking, what can I get here?” (P1)*

*“No, never received. They have never suggested anything … we’ll just go to the centre if we have any problem, for example when I ask them regarding the nearest PDK (Community-based center), then only they let me know the place.” (P4)*

*“I’m still hoping for help in terms of … er … help in term of special school. Yes, it is because there is no special center for CP child yet … for now. Searching from the website and then … actually like welfare … the procedures also … are complex. I send her to private school now.” (P7)*

#### 3.2.3. Emotional and Spiritual Support

Emotional issues were expressed as one of the great challenges faced by the parents in taking care of their children with CP. They feel stressed with the slow progress of their child despite attending many sessions of intervention. The parents constantly mentioned that they need emotional and spiritual support through counselling session to keep them strong in the journey of taking care of their child. However, the need was not fulfilled as such services were not being provided and easily available.

*“First challenge is … a major one, is actually emotion.I myself want to accept my child from normal to OKU [disabled] … [it] takes time … so counselling should be available. Not just for me but my child … that’s why I said special school isn’t available and counselling session for herself is not available yet. Counselling session for children … CP children … in regards to her condition … how she wants to accept herself.” (P1)*

*“Of course … until a point in time … like three to four months … when you already did (therapy) for a period of time but you do not see any result, you will feel down. Er … suppose to offer a counselling session. Because it’s very stressful right …” (P7)*

*“Every time … in the community center, I always ask, “Is there any course … talk … exposure which could boost my spirit … for me to always be in high spirits.”. Because this kind of thing … when we couldn’t achieve what we want up to a certain time, we will easily give up … but … no counselling service available. I think every parent would surely want counselling right? Because sometimes we … want to take care of him … we need to be mentally strong. We have to … our emotion have to be stable right? So I think (counselling) needed about one or two months once …” (P2)*

#### 3.2.4. Financial Support

Parents expressed that they need help in the financial aspect, especially in getting special equipment for their child as the equipment is expensive. Currently, financial support is limited and most parents are forced to have savings or to work extra hours for this purpose, which are difficult tasks. They somehow were relieved that they do not have to pay for therapy at government facilities.

*“None of her rehab equipment is cheap … just like that day … I bought that standing frame at the price of RM2600. I … actually our situation … actually we need more sources of money but for now I try to work from home. Luckily we come here (physiotherapy) for free of charge.” (P1)*

*“Daily expenses is still ok … the thing that he needs really … for example like the AFO or his chair … his equipment … those are expensive.” (P6)*

*“If anybody wants to take care of CP child right … they … use a lot of money … to buy her equipment is also expensive.” (P7)*

*“Collect K’s money … K’s allowance right … save day by day then only can buy (equipment).” (P8)*

*“Keep our own money … collect our own money too … only then we could buy the thing (equipment) if we want to.” (P9)*

#### 3.2.5. Explaining Child’s Condition to Others

The parents find it challenging for them to explain their child’s condition to others, especially to strangers. One of the reasons is limited understanding about CP among parents. Some parents decided to avoid meeting up with others in order to reduce this challenge.

*“His siblings were trained since young because I explain every day. I will explain about their brother’s condition … like this … so they need to be like this … For strangers … it is difficult (to explain).” (P2)*

*“The first challenge is … er … initially I felt ashamed because I have a special child … like that. To my other child, the sister … when she started schooling then we would talk slowly about A … now she already understands more and she could accept the fact and she … helps a lot …” (P3)*

*“I started telling them regarding, “I have such sibling like this. Hope they can accept. Yes, sometimes I don’t know how to explain. I don’t feel like I want to explain … Not that we feel shame…meaning…how do we explain. I just don’t want to explain … if can, better avoid meeting up. Because I don’t know how to explain, just say that his brain is injured … so physical movement isn’t so good.” (P6)*

*“For the family, they all understand…already accepted how their sibling is … I don’t know how to explain to others … I want to explain … if others ask about his disease … I don’t understand it so much. I mean if somebody asks … development delay … CP. What is CP? Don’t know.” (P8)*

*“When we go for a vacation right … or we meet with strangers, “Eh a grown-up already but still need to be carried.” So we feel … ah … we don’t know how to explain.” (P1)*

#### 3.2.6. Family Functioning

The parents have difficulty to involve their children with CP in outdoor activities because of their disability. They need to ensure the environment is safe and the facilities are disabled-friendly. One parent claimed that she stopped all travel plans except for travelling back to her hometown.

*“Difficult to bring her … to carry … bathing in the public toilet with her in kneeling position … and the toilet which we are not sure about its cleanliness … Actually, I have stopped all travel plans for now because we know that we will … if we went on a vacation, we will not be happy. So I stopped (travel plan), I avoid vacations. I only go back to our hometown.” (P1)*

*“Some of the event I am unable to bring N because of her condition. She must be in a comfortable state, if it’s too hot then she cannot. So I need to consider all these factors in order to decide whether to bring her along.” (P3)*

*“Ah … it’s difficult because of his condition lah. He … can’t go to any cold place, or strong wind too … I have to see how the environment is … I have to see where the location is … because it’s dangerous right? I need to think then only I can go.” (P2)*

In terms of the assignment of household chores, the family members always help each other in taking care of the child with CP. Lack of time for self after the birth of their child with CP is also one of the concerns among parents.

*“Er … we practise this … like, helping each other. I will take the responsibility to take care of her when the helper does the house chores. My wife is also working and she also need to cook, right … So we help each other. Her sister could already help the father with the clothes.” (P3)*

*“If my husband and I have job to be done outside, I asked for help from my eldest son, her brother is already 14 years old so he understands … He is very understanding about how his sister is, so I asked him to help to take care of the sister for 2 to 3 hours.” (P5)*

*“Sometimes … exactly yes (need time for own self) … but there is none.” (P1)*

*“No, I do not have time for my own self.” (P2)*

## 4. Discussion

To the best of our knowledge, this is the first qualitative study to explore and discuss the needs and unmet needs of parents of highly dependent children with CP in a Malaysian setting. Our findings from this study suggest various aspects of the parent’s needs are still unmet despite the advance of our healthcare system.

The need for information appears to be the most frequently reported, as highlighted by other studies [6,7,8,10]. The participants of our study reported that they have needs in seeking various kinds of information regarding their child, but those needs were fulfilled mostly through the internet or parents of other children with CP. The participants claimed that the information received from the healthcare professionals was not conveyed in the way they desire, for example, the information was either difficult to understand or is inadequately provided in the primary healthcare centre. Eventually, when all the needs in this aspect were satisfied, it was by the means of their own initiative. Over time, the parents become more knowledgeable regarding their child, so their needs evolve [14].

In terms of social support, the need for support from a counsellor for themselves and for the siblings of children with CP was unmet. This might be due to the lack of such services by related agencies. Additionally, most parents are also not comfortable to seek help because of social stigma [22], whereby these parents suffer from low self-esteem and are anxious of being judged negatively, which consequently became a barrier to treatment [23].

None of the participants had a problem in locating a day care centre and getting welfare for daily necessities for their children although these services were received through great difficulties. The need for special schooling was highly expressed by the participants in our study. One possible explanation for this is due to the limited number of special education schools or classes in public schools which emphasise academic performance [10]. Furthermore, the participants reported unmet needs in terms of sponsorship for special equipment. This need is apparent due to the fact that the children in our study are highly dependent, thus are less mobile and have greater needs for special equipment. It can be hypothesized that equipment for children with special needs in Malaysia is expensive due to the limited number of local manufacturers hence high dependency on imported equipment.

The need for financial support in purchasing special equipment is vital as they do not get any sponsorship. However, their need in paying for medical and rehabilitation fees were fulfilled which is in contrast to overseas studies because the children with CP in Malaysia are excluded from paying medical and rehabilitation fees if they seek treatment from government hospitals. On the other hand, parents who opt to visit a private medical centre for intensive and frequent therapy need to spend a minimum of RM 29,000 per year [24].

The parents in our study reported that their need in explaining the child’s condition to family members and other children was contented although challenging. However, the need in explaining the condition to strangers was not attained. There are parents who claimed that they initially felt ashamed, and this may be due to the concern of being rejected by the society due to the stigma associated with children with disability [25].

For family functioning, all the needs for internal family functioning were achieved. This is possible because, typically, people do not seek help to solve internal problems but rather exercise self-control [26]. The need for outdoor activities including leisure was highly reported by the parents who claim that finding suitable activities and facilities is inconvenient to them. This illustrates a low participation level of the children with CP. The results are supported by the findings of past studies that children with CP have low satisfaction with recreational services [27] and very limited number of friends to play with [28].

Strategies to address the unmet needs of these families are urgently needed. One provision that can give an advantage to the families is opportunity for engagement with counsellors. Active engagement with counsellors will enable the parents to effectively adjust to a wide variety of challenging roles, hence reducing their stress level and needs for psychological and spiritual support [29]. It is recommended that family conference sessions which are normally organized by the pediatric department in hospitals could include a health psychology to address the needs for counselling among the parents.

Participation in the community environment is important for wellbeing, promoting a sense of belonging and opportunities for social networks and physical activity [30]. Participation of the children with CP in outdoor activities can be facilitated by getting health care professionals to play a role in planning suitable community activities for them. Outdoor activities for more-abled child with CP as organised in several community-based rehabilitation centers [31] could be modified to suit the highly dependent children with CP. The role of other related agencies such as social welfare department and local city council is also recommended to facilitate successful participation among the children. In addition, participation could be promoted in suitable setting in special schools. Issues regarding inadequacy of the special education system for children with CP demonstrates a necessity for the Government to formulate a solution towards meeting this need in the future.

Our study has several limitations. Firstly, it only reflects the needs of families who were already receiving rehabilitation and day care center services. The needs and unmet needs of families who do not receive such services remain unknown. The wide age range of children (i.e., between 4 and 15 years) in this study might also impact the results of the interview as the unmet of caregivers would be different depending on their child’s age. Next, the majority of the participants are young parents who are highly educated and live in urban areas. The parents’ needs might be different than those of older or less educated parents from rural areas. The majority of the participants (parents) in this study were females (mother); this is because mothers are the main caregivers for the children in our study. Finally, our sample size is smaller than the recommended size for an interview. Some of the participants were also less expressive although prompted to share more during the interview. These caused difficulties in ensuring data saturation.

Despite these limitations, the study not only provided information regarding the needs and unmet needs of families of highly dependent children with CP, but also reflects a clear picture on how the needs are fulfilled and how the unmet needs are coped with.

## 5. Conclusions

In conclusion, the unmet needs of parents of highly dependent children with CP include the need for receiving information regarding CP, getting emotional and financial support, explaining the child’s condition to strangers, support from social welfare department and family functioning. Since this is the first qualitative study done in Malaysia, the results of this study will help healthcare professionals and related agencies to develop appropriate strategies and interventions for the parents of children with CP. Further study is needed to strengthen the study’s findings and should include participants who do not receive any rehabilitation and day care service, including those living in rural areas.

## Figures and Tables

**Table 1:** Profiles of parents and children with cerebral palsy (CP) (*n* = 9).

Code	Gender	Job	Education Level	Age	Child’s Age	Siblings	Family Background	Child’s Cognitive *	Child’s Comorbidities	GMFCS
P1	Female	Teacher	Tertiary	37	4	3rd out of 3	Both parents supportive	Yes	Yes ^#^	Level IV
P2	Female	Government servant	Tertiary	32	7	1st out of 2	Both parents supportive	No	No	Level III
P3	Male	Officer	Tertiary	35	7	2nd out of 2	Both parents supportive	Yes	No	Level IV
P4	Female	Businesswoman	Tertiary	40	15	2nd out of 3	Both parents supportive	Yes	No	Level V
P5	Female	Housewife	Tertiary	34	12	2nd out of 2	Both parents supportive	Yes	No	Level IV
P6	Female	Government servant	Tertiary	33	5	Only child	Both parents supportive	Yes	No	Level IV
P7	Female	Housewife	Tertiary	42	12	Only child	Both parents supportive	Yes	Yes ^#^	Level IV
P8	Female	Housewife	Tertiary	51	9	2nd out of 3	Both parents supportive	Yes	No	Level V
P9	Female	Housewife	Secondary	37	6	Only child	Both parents supportive	Yes	Yes ^#^	Level V

* Follows simple command. ^#^ Epilepsy. GMFCS: gross motor classification function score.

## References

[1] Lin S.L. (2006). Coping and adaptation in families of children with cerebral palsy. Except Child..

[2] King S., Teplicky R., King G., Rosenbaum P. (2004). Family-centered service for children with cerebral palsy and their families: A review of the literature. Semin. Pediatr. Neurol..

[3] Wood E., Rosenbaum P. (2000). The gross motor function classification system for cerebral palsy: A study of reliability and stability over time. Dev. Med. Child. Neurol..

[4] Liptak G.S., O’Donnell M., Conaway M., Chumlea W.C., Worley G., Henderson R.C., Calvert R. (2001). Health status of children with moderate to severe cerebral palsy. Dev. Med. Child. Neurol..

[5] Almasri N.A., Palisano R.J., Dunst C.J., Chiarello L.A., O’Neil M.E., Polansky M. (2011). Determinants of needs of families of children and youth with cerebral palsy. Child. Health Care.

[6] Bertule D., Vetra A. (2014). The family needs of parents of preschool children with cerebral palsy: The impact of child’s gross motor and communications functions. Medicina.

[7] Nitta O., Taneda A., Nakajima K., Surya J. (2005). The relationship between the disabilities of school-aged children with cerebral palsy and their family needs. J. Phys. Ther. Sci..

[8] Palisano R., Almarsi N., Chiarello L., Orlin M., Bagley A., Maggs J. (2010). Family needs of parents of children and youth with cerebral palsy. Child. Care Health Dev..

[9] Wang P., Michaels C.A. (2009). Chinese families of children with severe disabilities: Family needs and available support. Res. Pract. Pers. Sev. Disabil..

[10] Tan S.H. (2017). Assessing the needs of caregivers of children with disabilities in Penang, Malaysia. Health Soc. Care Community.

[11] Suriati S., Zainiyah S., Lye M., Norlijah O. (2011). Assessing the unmet needs among caregivers of children with disabilities at the community-based rehabilitation centres in Selangor. Malays. J. Public Health Med..

[12] Carpenter B. (2000). Sustaining the family: Meeting the needs of families of children with disabilities. Br. J. Spec. Educ..

[13] Ellis J.T., Luiselli J.K., Amirault D., Byrne S., O’Malley-Cannon B., Taras M., Sisson R.W. (2002). Families of children with developmental disabilities: Assessment and comparison of self-reported needs in relation to situational variables. J. Dev. Phys. Disabil..

[14] Kruijsen-Terpstra A.J., Verschuren O., Ketelaar M., Riedijk L., Gorter J.W., Jongmans M.J., on behalf of the Commonwealth Neuroendocrine Tumour Collaboration (CommNETs) (2016). Parents’ experiences and needs regarding physical and occupational therapy for their young children with cerebral palsy. Res. Dev. Disabil..

[15] Hornby A.S., Wehmeier S. (1995). Oxford Advanced Learner’s Dictionary.

[16] Crouch M., McKenzie H. (2006). The logic of small samples in interview-based qualitative research. Soc. Sci. Info..

[17] Guion L.A., Diehl D.C., McDonald D. (2001). Conducting an In-Depth Interview.

[18] Bailey D.B., Simeonsson R.J. (1988). Assessing needs of families with handicapped infants. J. Spec. Educ..

[19] Guest G., Bunce A., Johnson L. (2006). How many interviews are enough? An experiment with data saturation and variability. Field Methods.

[20] Thurmond V.A. (2001). The point of triangulation. J. Nurs. Scholarsh..

[21] Bekhet A.K., Zauszniewski J.A. (2012). Methodological triangulation: An approach to understanding data. Nurse Res..

[22] Vogel D.L., Wester S.R., Larson L.M. (2007). Avoidance of counseling: Psychological factors that inhibit seeking help. J. Couns. Dev..

[23] Cantwell J., Muldoon O., Gallagher S. (2015). The influence of self-esteem and social support on the relationship between stigma and depressive symptomology in parents caring for children with intellectual disabilities. J. Intell. Disabil. Res..

[24] Kamaralzaman S., Ying T.C., Mohamed S., Toran H., Satari N., Abdullah N. (2018). The economic burden of families of children with cerebral palsy in Malaysia. Malays. J. Public Health Med..

[25] McCabe H. (2007). Parent advocacy in the face of adversity: Autism and families in the People’s Republic of China. Focus Autism Other Dev. Disabl..

[26] Sivabalan T., Zakaria E., Amin A.S. (2018). Exploring Coping Strategies of Mother in Taking Care of a Child with Cerebral Palsy Disability). Akademika.

[27] Magill-Evans J., Darrah J., Adkins R. (2003). Youths with cerebral palsy and their satisfaction with recreational services: Implications for inclusion. Leis. Loisir..

[28] Joginder Singh S., Iacono T., Gray K.M. (2014). An investigation of the intentional communication and symbolic play skills of children with down syndrome and cerebral palsy in Malaysia. J. Early Interv..

[29] Oluremi D. (2015). Counselling Intervention and Support Programmes for Families of Children with Special Educational Needs. J. Educ. Pract..

[30] Carroll P., Witten K., Calder-Dawe O., Smith M., Kearns R., Asiasiga L., Kayes N., Mavoa S. (2018). Enabling participation for disabled young people: Study protocol. BMC Public Health.

[31] Jaafar N.A., Hasan H., Mohd Nordin N.A., Aljunid S.M. (2018). Cost effectiveness of community—Based rehabilitation (CBR) for children with disability. Malays. J. Public Health Med..

